# A Randomized-Controlled Trial of Mentalization-Based Treatment Compared With Structured Case Management for Borderline Personality Disorder in a Mainstream Public Health Service

**DOI:** 10.3389/fpsyt.2020.561916

**Published:** 2020-11-12

**Authors:** Dave Carlyle, Robert Green, Maree Inder, Richard Porter, Marie Crowe, Roger Mulder, Chris Frampton

**Affiliations:** ^1^Department Psychological Medicine, University of Otago, Christchurch, New Zealand; ^2^Canterbury District Health Board, Mental Health Services, Christchurch, New Zealand; ^3^Department Psychological Medicine, University of Otago, Christchurch, New Zealand

**Keywords:** borderline personality disorder, mentalization, enhanced therapeutic case management, public health service, psychotherapy

## Abstract

**Background:** Treatment of borderline personality disorder (BPD) in publicly funded mental health services generally use approaches based on medical interventions and generic case management. Specific psychological therapies developed for BPD may be more effective but have rarely been evaluated in routine clinical practice.

**Aim:** To examine the effectiveness of Mentalization Based Treatment (MBT) in adults with an established diagnosis of BPD under the care of a publicly funded Mental Health Service (MHS), on rates of non-suicidal self-harm (NSSH) and attempted suicide (SA).

**Methods:** A randomized, controlled trial (RCT) comparing 18 months of MBT with Enhanced Therapeutic Case Management (ETCM), a form of Structured Clinical Case Management (ICTRP: ACTRN12612000951853). Participants were adults recruited from a patient population under the care of a publicly funded mental health service (MHS) with a confirmed diagnosis of BPD. The primary outcome measures were the incidence of non-suicidal self-harm or suicide attempt over 18 months of treatment.

**Results:** 72 participants (71 females, 1 male) were randomized to MBT (*n* = 38) or ETCM (*n* = 34). Both groups showed a significant reduction in the overall incident rate of SA and NSSH. Between groups, SA rates were higher in the MBT group and conversely NSSH rates were higher in the ECTM group.

**Conclusions:** The introduction of a structured service that delivered a structured psychotherapy (MBT) and an effective case management approach (ETCM) both resulted in a reduction in SA and NSSH. The differences in improvements found between groups within this study setting will require further research.

## Introduction

BPD is a serious disorder affecting 1–2% of the general population, up to 10% of psychiatric outpatients and 20% of in-patients (1). Pervasive difficulties with emotion regulation, impulse control, and instability in relationships and self-image result in high use of mental health services (2, 3). Non-suicidal self-harm is a feature which is distressing to patients and their families, results in significant morbidity and has high costs to the health service (4).

A widening range of treatment approaches demonstrate efficacy in addressing key features of the disorder (self-harm, suicidality, high service use, etc.). Evidence suggests that Dialectical Behavior Therapy, Mentalization-Based Treatment, Transference-Focussed Therapy and Schema-Focussed Therapy are effective in reducing BPD symptoms including self-mutilation, suicide attempts, anxiety and depression, hospitalization and impaired social functioning (5, 6). Additionally, well-structured clinical care that is sympathetic to the unique interpersonal problems of people with BPD (MBT and DBT) has also been shown to be effective in reducing symptom distress (7, 8).

Although efficacious interventions have been identified, results obtained in optimal research environments, using skilled therapists, often do not translate to everyday clinical situations where therapists rely on distributed training (9, 10). In BPD, the translation of positive clinical trials into everyday clinical services has not been adequately addressed. Studies by McMain et al. (11) and Feigenbaum et al. (12) confirm the utility of DBT in real-world clinical settings. However, others have highlighted the shortcomings and barriers to implementation such as the burden of training and the impact of this on staff recruitment. The time commitment of the DBT model was also identified as an issue in the establishment and maintenance of this in mainstream clinical practice (13).

Evidence for the generalisability of MBT into everyday practice is also equivocal at present. A recent Dutch study replicated the initial results of Bateman and Fonagy (14) for the day hospital form of MBT (MBT-DH) and demonstrated that MBT-DH translates well into everyday clinical settings (15, 16). Positive results in a Danish clinic for personality disorders and a Norwegian mental health clinic, suggest that the outpatient form of MBT (MBT-OP) seems likely to be translatable into everyday clinical settings (17, 18). However, Jorgensen et al. did not report on the effects of treatment on self-harm, suicidal behavior/ideation or service use, important outcomes in this condition, and the Norwegian study was a naturalistic longitudinal evaluation with no randomization.

In 2006, The New Zealand Mental Health and Addiction Action Plan 2006–2015 (Te Kokiri) (19) encouraged the publicly-funded mental health services in New Zealand to develop service frameworks supporting wider access to psychological therapies for all users, particularly individuals with severe personality disorder. The Mental Health Division of the Canterbury District Health Board took advantage of the additional resources provided to introduce a specialist treatment service for BPD within existing community mental health teams. Development of a specific MBT service for the treatment of BPD within a publicly funded Mental Health Service (MHS) began in September 2009. Until this time, no specific treatment service for adults with BPD was available within this DHB.

The introduction of a new service provided an opportunity to examine whether the results of the Bateman and Fonagy (7) MBT-OP study could be replicated in a different country, with generic mental health professionals under *real-world* training/supervision constraints, treating usual clients of secondary-level services. In replicating the Bateman and Fonagy (7) study the same primary outcome measures were used: crisis events - suicide, suicide attempt and self-harm. In this study, subjects were recruited from existing service caseloads and normal service referral processes. Many patients with BPD had been engaged with the MHS long term, with increasing severity of symptoms evident from a review of case files pre-entry.

## Methods

### Trial Design

This RCT was designed to evaluate the effect of 18 months of structured MBT treatment compared with ETCM in an outpatient setting in a publicly funded MHS for people with BPD. Primary outcomes were rates of non-suicidal self-harm and attempted suicide.

The authors assert that all procedures contributing to this work comply with the ethical standards of the relevant national and institutional committees on human experimentation and with the Helsinki Declaration of 1975, as revised in 2008. All procedures involving human subjects/patients were approved by the Southern Regional Ethics Committee, Ministry of Health, NZ: URB/09/06/026.

This RCT was registered with the International Clinical Trials Registry Platform (ICTRP): ACTRN12612000951853.

### Subjects

Subjects were existing patients of the community mental health service, with a diagnosis of BPD, confirmed by the SCID-II. Written informed consent was obtained from all subjects/patients.

Exclusion criteria were kept to a minimum and were diagnoses of psychoses or primary substance dependence that interfered with treatment engagement, insufficient proficiency in English to engage in psychotherapy, and concurrent engagement in a structured psychological treatment for personality disorder.

### Therapists

Mental health clinicians (psychologists, nurses and social workers) in existing community mental health teams delivered MBT. All therapists received training in MBT prior to the beginning of the service start-up. Treatment adherence was supported through fortnightly individual supervision from a local MBT supervisor and monitored through fortnightly group supervision Skype sessions with Prof Anthony Bateman. The control group received an enhanced standard of outpatient care that followed the guidelines for SCM employed in the Bateman and Fonagy study (20).

### Interventions

MBT is a time-limited therapy of structured interventions that aim to remediate the deficits in social cognition (mentalizing) that have been observed in BPD (21, 22) and which are postulated to underlie the central difficulty of emotion regulation (23). The intervention group received 18 months of MBT psychotherapy consisting of a 1-h individual therapy session and a one and half hour group therapy session each week. The first 12 weeks of the group treatment consisted of a structured education programme on BPD and mentalization to provide participants with a standard level of knowledge regarding the disorder and the treatment approach. The remaining 15 months of group work involved a modified group analytic approach that supported individuals to retain a reflective approach within interpersonal relationships occurring in the group and in their review, in the group of recent life experiences. A case manager (CM) developed a treatment and crisis plan with each participant and provided crisis intervention support as needed.

As in the Bateman and Fonagy (7) study, the 18-month control condition received a higher level of clinical care than treatment as usual (TAU) in order to provide a strong test of the model's effectiveness. Case managers for the ETCM control group received training using the now published manual for Structured Clinical Management (SCM) (24) that included specific knowledge of the psychological difficulties in BPD, treatment principles for structured care, treatment and crisis planning, therapeutic relationship skills, and specific interventions addressing problem-solving and managing self-harm. A range of experienced mental health staff comprised of nurses, social workers and occupational therapists delivered ETCM.

Participants in both conditions retained access to a psychiatrist for the purposes of review and prescribing of medications, as required.

Because MBT and ETCM involved different treatment conditions, participants and clinicians were unable to be blind to which group each participant was assigned. Control group participants were aware they could access MBT treatment following the completion of the study should ETCM not assist them.

### Randomization

Randomization was undertaken utilizing a computer-generated sequence in permuted blocks of four. An independent administrator held the randomization code.

### Outcome Measures

Outcomes were monitored from 0 to 18 months participation in the study. Specific data on these outcomes were obtained through reports generated from integrated electronic health records. This electronic database captures and records all medical and psychiatric presentations, pertinent clinical data and patient details. Case notes were tracked to recover details of presentations, nature of harm, interventions required and outcome of event. Presentations were accessed from medical records for secondary services. Access to data on primary care (general practice) medical attention could not be obtained. However, it was considered that most of the presentations of a serious nature were likely to be to the hospital emergency department (ED).

### Primary Outcome Measures

Episodes of deliberate self-harm were assessed by detailed consideration of the medical notes by a rater who was blind to treatment assessment. These events were then coded as either

Non suicidal self-harm (NSSH) - defined as an event serious in nature which required medical assessment and intervention but in which there did not appear to have significant suicidal intent or -

Suicide attempts (SA) - defined as events which were serious in nature and required medical assessment and intervention.

### Statistical Power

The sample size of 72 patients in total (36 MBT/36 ETCM) was required to detect as statistically significant (2-tailed α = 0.05) with 80% power, a higher percentage of patients refraining from SA/NSSH in the MBT group of 65% compared with 35% in the ETCM group, over an 18 month period. These estimates are based on findings from Lorentzen et al. (25) and Bateman and Fonagy (20).

### Data Analysis

Analyses were conducted using the Statistical Package for the Social Sciences (SPSS) version 23 for Windows. Descriptive analyses including frequencies, percentages, means and standard deviations (SD) were used to describe the demographic and clinical characteristics of the MBT and ETCM groups. Means, ranges and SD were used to summarize the number of individual MBT and ETCM sessions over the 18-month treatment period and the number of MBT group sessions.

Episodes of NSSH/SA were analyzed for all patients initially randomized using a chi-square test. These results are shown graphically using histograms in **Figures 2**–**4**. Supporting analyses were undertaken comparing the rates of new admissions as the number of SA and NSSH events per person-month, using a Poisson approximation. Rates of SA and NSSH were calculated over the 18 month period.

Additional analyses were undertaken to compare rates of SA and NSSH at each 6 month period; 0–6 months, 6–12 months, and 12–18 months also using the Poisson approximation.

## Results

### Recruitment

**Figure 1:**
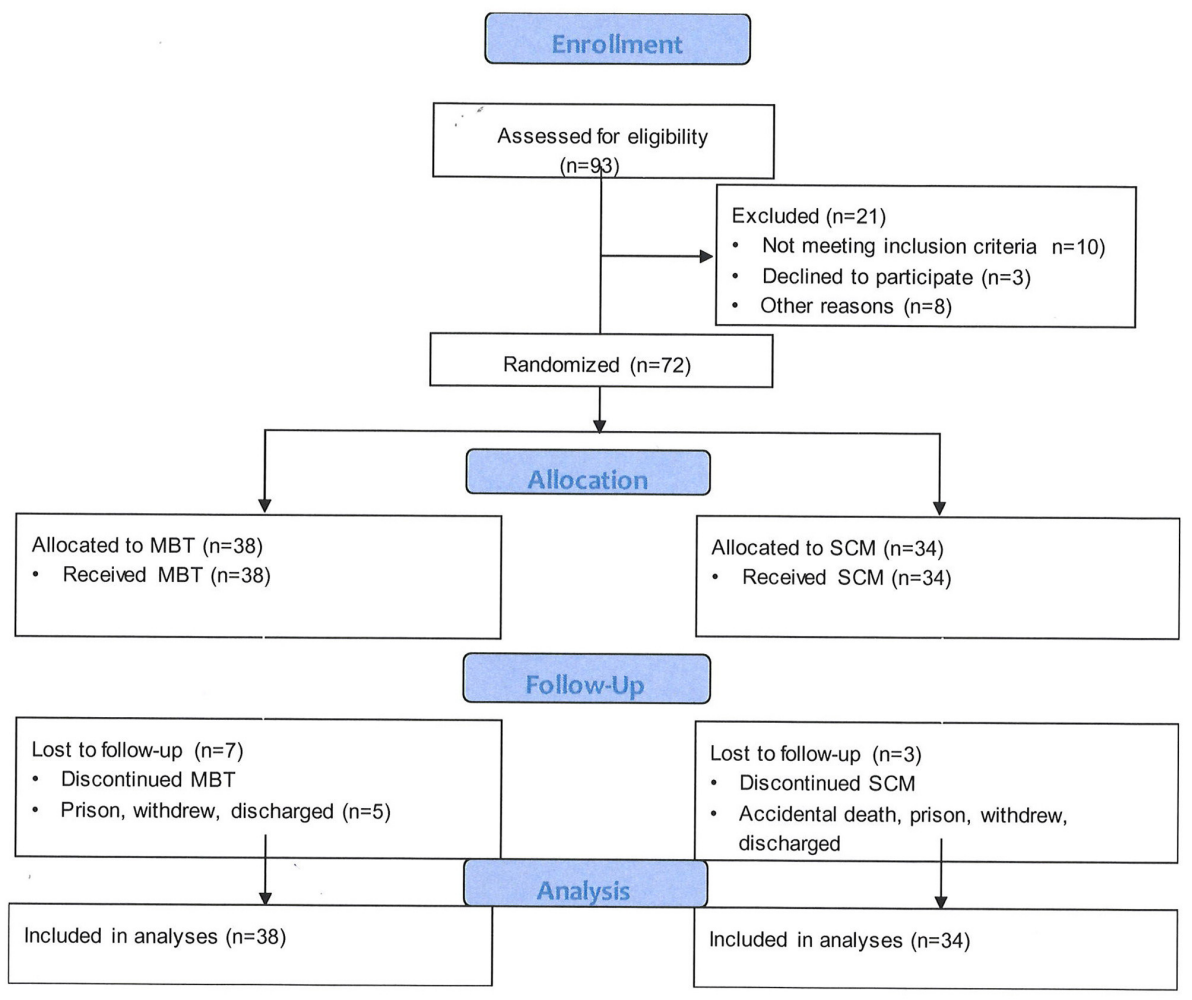
Patient progression through a randomized-controlled trial of Mentalization-Based Treatment compared with structured case management for borderline personality in a mainstream public health service.

Figure 1 shows the study participant flow through the trial. Ninety-three outpatients of the community mental health service, with a possible diagnosis of BPD were identified for screening for inclusion. Of these potential participants, 10 did not meet criteria for entry due to other active primary diagnoses or current hospitalization. Three declined to participate and eight did not participate for other reasons such as leaving the region at time of entry.

Seventy-two patients with a diagnosis of BPD were recruited consecutively from 1st September 2009. Recruitment was stopped in October 2011 when referrals to the service were not sufficient to form coherent intakes to establish functional therapy groups. From September 2011, the city experienced a series of devastating earthquakes, which prevented further recruitment. The final participants completed the treatment course in late 2013.

### Subject Characteristics

The baseline demographic characteristics are presented in Table 1. The mean number of Borderline Personality Disorder criteria was similar for the two groups with 7.6 (SD 1.4) in the MBT group and 7.2 (SD 1.4) in the ETCM group. In the 6 months prior to entry 15 people in MBT group made 39 SA compared with 15 in the ETCM group having 35 SA. For NSSH MBT group had 9 people had 29 incidents and in ETCM 10 people had 15 events.

**Table 1:** Demographic and clinical characteristics of the MBT and ETCM groups.

	**MBT *n* = 38**	**ETCM *n* = 34**
Age mean (SD)	32.4 (9.8)	31.6 (11.7)
Gender -female n (%)	38 (100.0)	33 (97.1)
**Ethnicity n (%)**		
NZ European	30 (78.9)	27 (79.4)
Maori	2 (5.3)	2 (5.9)
Other	1 (2.6)	1 (2.9)
European other	5 (13.2)	4 (11.8)
Baseline GAF score mean (SD)	45.4 (9.2)	43.5 (10.26)
6 months pre-baseline history of suicide attempt n (%)	19 (50)	19 (55.9)
6 months pre-baseline history self-harm n (%)	12 (31.6)	14 (41.2)
**Axis II comorbidity n (%)**		
Avoidant PD	23 (60.5)	23 (67.6)
Dependent PD	4 (10.5)	6 (17.6)
Obsessive compulsive PD	9 (23.7)	6 (17.6)
Paranoid PD	9 (23.7)	8 (23.5)
Schizotypal PD	2 (5.3)	1 (2.9)
Schizoid PD	0	1 (2.9)
Histrionic PD	1 (2.6)	0
Narcissistic PD	1 (2.6)	0

### Outcomes

There was no difference in the percentage of patients who abstained from NSSH over 18 months -MBT *n* = 26 (68%), ETCM *n* = 17 (50%) (χ^2^ = 2.62, *p* = 0.11) or in the percentage engaging in SA – MBT *n* = 19 (50%), ECTM *n* = 18 (53%) (χ^2^ = 0.06, *p* = 0.81).

**Figure 2:**
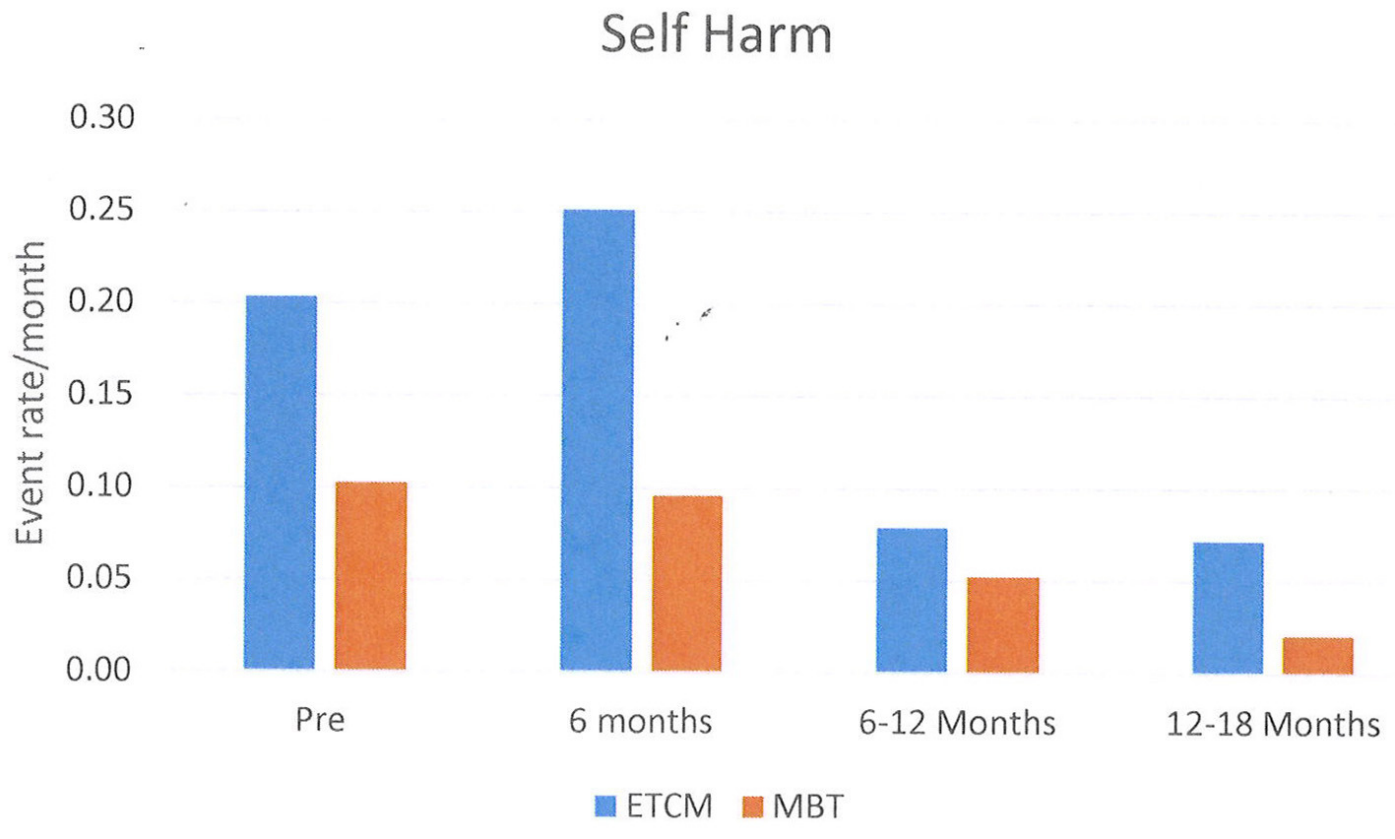
Rates of Non-suicidal Self Harm per month shown in 6 month blocks.

The rates of NSSH per patient month were 0.09 in the MBT group and 0.22 in the ETCM group. The rate ratio for NSSH over the total 18 month period was 0.43, 95% CI [0.30, 0.59), statistically significantly higher in the ETCM group (*p* < 0.001) (See Figure 2).

**Figure 3:**
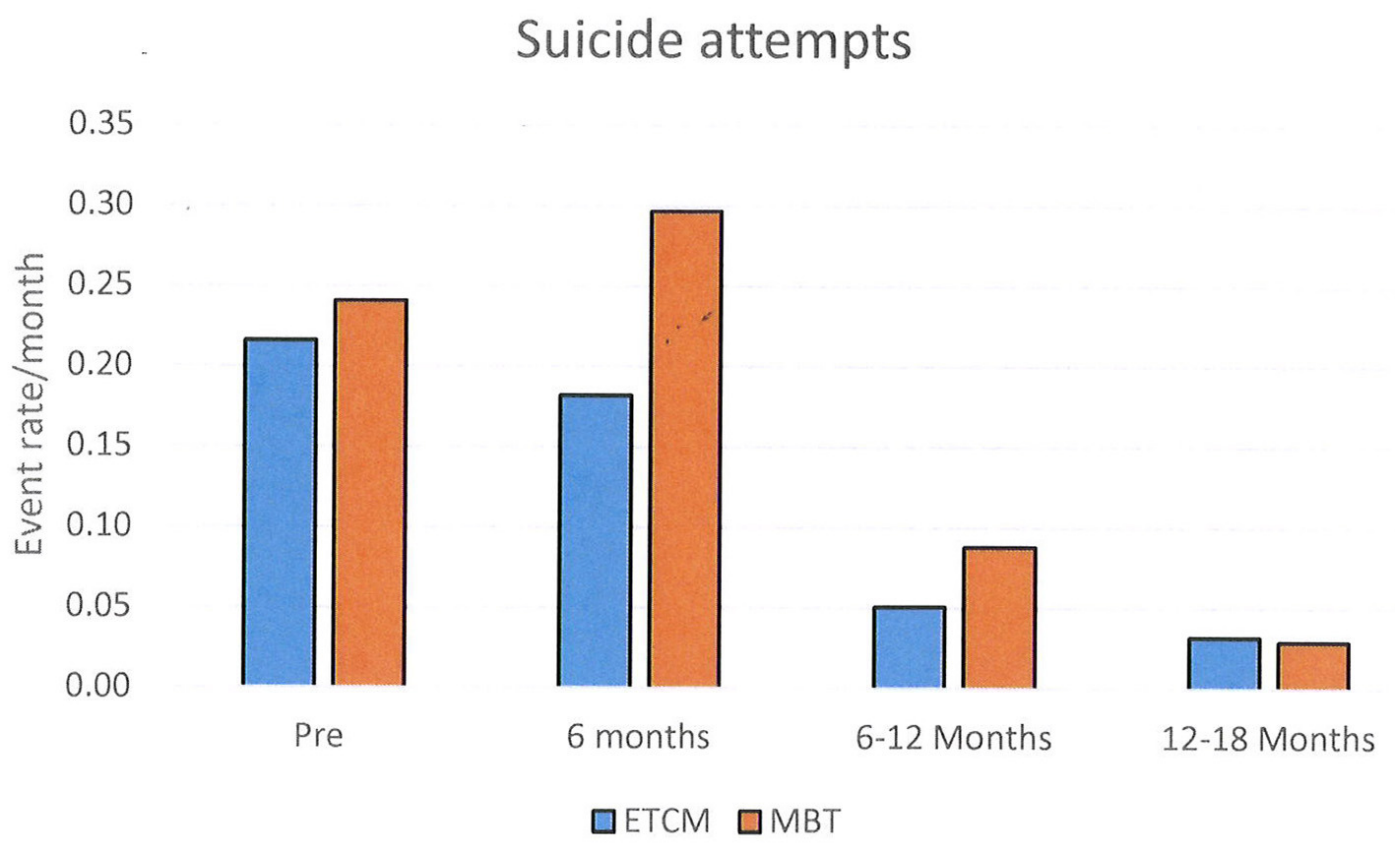
Rates of Suicide Attempt per month shown in 6 month blocks.

The rates of SA were 0.20 in the MBT group and 0.14 in the ETCM group. The rate ratio for SA over the total 18 month period was 1.48, 95% CI (1.09, 2.02) which was statistically significantly higher in the MBT group (*p* = 0.009) (See Figure 3).

**Figure 4:**
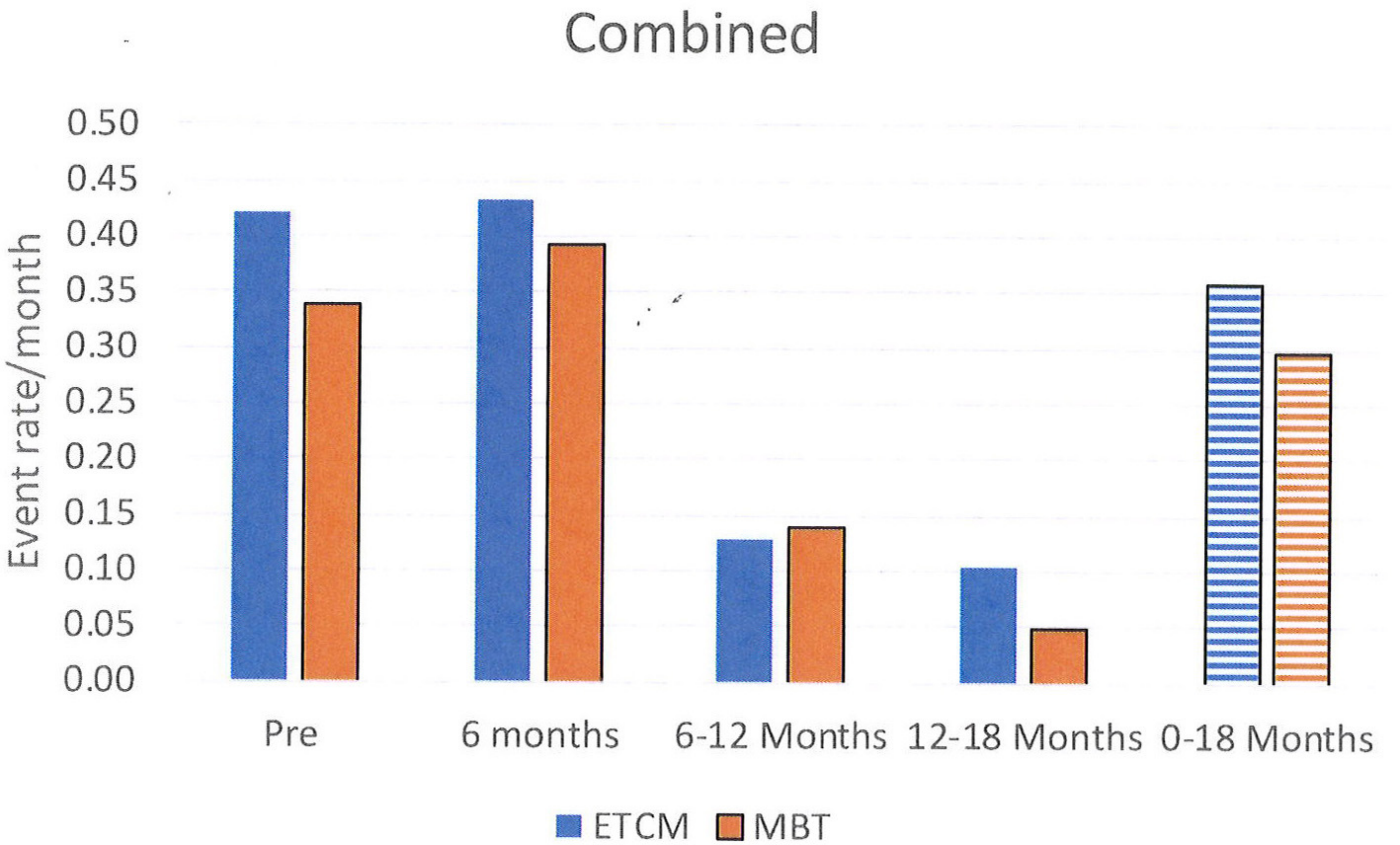
Rates of all self-harm, combining SA, and NSSH, per month shown in 6 month blocks and total over 18 months.

Rates of all self-harm (SA and NSSH combined) were 0.30 in the MBT group and 0.36 in the ETCM group. The rate ratio for SA over the total 18 month period was 0.83, 95% CI (0.67, 1.03), *p* = 0.08 (See Figure 4).

Between the 6 months pre-baseline and the final 6 months of therapy, monthly rates of NSSH reduced significantly: pre baseline – 0.15, 12–18 months – 0.04, rate ratio was 0.29, 95% CI (0.19, 0.44), *p* < 0.001. Monthly rates of SA also reduced significantly: pre baseline- 0.23, 12–18 months – 0.03, rate ratio was 0.13, 95% CI (0.09, 0.20), *p* < 0.001. Reductions in both groups were not evident until 6–12 months after the intervention started and these reductions persisted for the following 12–18 month period.

### Secondary Outcomes

#### Service Use

**Figure 5:**
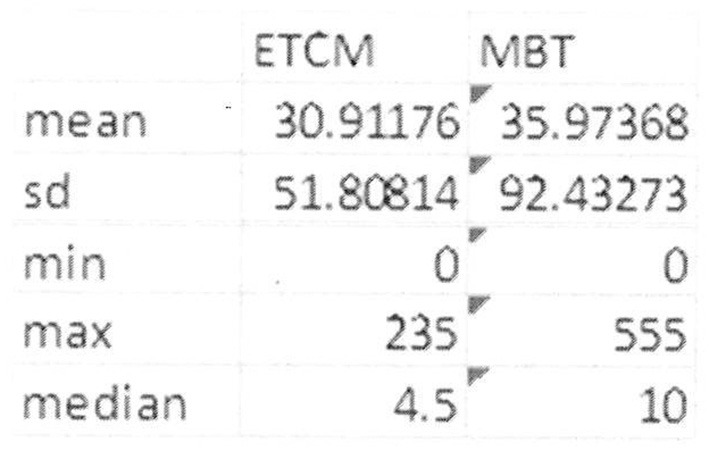
Inpatient service usage in bed days by participants in the course of the study showing range and median.

Admissions to adult inpatients services occurred in 47% (n = 17) of the MBT group with a range of duration of admission of 1–555 days. For the ETCM group 41.2% (*n* = 14) were admitted with a range of 3–235 days. There was no significant difference between groups in number of days admitted to inpatient units. The median for ETCM being 4.5 with a standard deviation of 51.808. The median for the MBT group being 10 with a SD of 92.432 (See Figure 5). Both groups were influenced by single outliers.

## Discussion

This was a pragmatic RCT of MBT for BPD in a public health system. The primary outcome, self-harm, was chosen since this is both practically and functionally important and data could be reliably obtained from hospital records, thereby minimizing data collection from patients. The main findings were that compared with a control therapy -ETCM, there was no difference in the percentage of patients who abstained from any form of self-harm over the subsequent 18 months.

When examining rates of self-harm, overall, there was no difference in self-harm between groups. Of interest, however, when self-harm was sub-divided into self-harm with serious suicidal intent (SA) and self-harm without serious suicidal intent (NSSH) there were differences between groups. SA rates were higher in the MBT group and conversely NSSH rates were higher in the ECTM group. The finding suggests that while rates of total self-harm were similar across groups, those receiving MBT were likely to engage in self-harm with more serious suicidal intent. As noted earlier, inpatient psychiatric hospitalization rates were not significantly different in either arm arms so this does not appear to bias outcomes of either group.

What is very significant is that total rates of SA and NSSH were greatly reduced, being halved, for both groups by the 12–18 month stage of both the interventions compared with the 6 months pre baseline when they had been receiving TAU. This is a very important finding of the study. While there was no overall significant advantage of MBT over ETCM, the reduction in both types of self-harm over 18 months suggests a positive effect of both compared with the previous treatment which patients received (medication management and non-structured case management) and adds some evidence to the positive studies of Bateman and Fonagy with day patient MBT (15, 16).

Longer-term comparison between groups (following the end of the programme) was not undertaken because those patients undergoing the ECTM were then given the opportunity to switch to the MBT programme so would have been in intensive therapy for a further 18 months.

The finding of marginally higher rates of SA in the MBT group is interesting, but it must be acknowledged that overall there was a large reduction in SA for both groups compared with baseline. Unfortunately, in this data there was no specific measurement of the physical consequences of the self-harm or the sequelae of this. One possible reason for the difference between groups is that those in the ETCM group generally stayed with a case manager with whom they already had an established relationship, whilst those in the MBT group moved to a new primary relationship with their newly allocated therapist. It is possible that the higher suicidal intent in the MBT group is a reaction to the loss of the attachment to the previous case manager. In support of this, the largest differences in rates are seen in the first 6 months, after which the rates decline significantly in both groups. Training regular case managers to undertake specific therapies for BPD rather than having separate specialist teams could mitigate such issues. Again the results need to be interpreted in the context of an overall reduction in NSSH and SA from pre-baseline levels, at least after the initial 6 months for both groups.

A Cochrane review of treatments for BPD (6) noted that, while the efficacy of DBT had been independently replicated, there remained a need for a similar evaluation of the efficacy of MBT. This study provides independent replication of the outpatient form of MBT and ETCM and its ability to clinically affect the key behavioral problems associated with BPD such as NSSH and SA.

This study also adds to the growing evidence base that good clinical care following manualised guidelines is effective (7, 17, 26, 27). Both structured therapies showed significant reductions in both types of self-harm from baseline to the last 6 months of treatment. This finding is consistent with McMain et al. (8, 11) who did not find an advantage for the active treatment (DBT) over manualised general care, though found an advantage of both compared with baseline. It is not clear at this point whether a particular structured therapy (MBT, DBT, or a general manualised therapy) has an advantage over any other. Further studies or meta-analyses, which overcome relatively low power, may answer this in future.

Several hypotheses for the lack of difference in outcome for MBT and ETCM require investigation. There is some evidence that comorbid avoidant traits are a negative prognostic factor (28). In a relatively small service, treatment leakage between the two groups is a factor, which can reduce differences. Although separate training of ETCM and MBT clinicians was undertaken, an additional group of “back-up” MBT therapists were trained in case of therapist loss. It was outside the control of the trial to prevent team leaders assigning these clinicians as case managers to clients in the study. We suspect that the additional mentalizing skills of these clinicians could have contributed to better outcomes in the ETCM arm. We also suspect that therapist inexperience may have contributed to a lower response in the MBT arm. Although we lacked at the time formal tools to rate therapist competence and fidelity, informal clinician and supervisor feedback indicates considerable likelihood that MBT therapists in the study may have operated differently in the 1st year of the study, compared with their facility in later years. As early intakes included existing patients with marked severity, it is likely that the combination of untested therapists and severely symptomatic patients may have had an impact on the effectiveness of the intervention. These issues are of course inherent in using psychological treatments in a public health system.

At the broadest level, this research confirmed that the introduction of a treatment service that followed manualised guidelines and focussed on the specific needs of people with BPD, resulted in significant improvements in key problem areas for both groups. There is a marked difference between the clinical journey of patients in the study before treatment, and their positive progress in systematic treatment. This improved outcome was not dependent on being a well-defined psychotherapy MBT model or a structured case management model as in ETCM. This study provides clear evidence that the new service is beneficial, providing clinically significant outcomes for clients. A further analysis of health service costs will be reported in a separate paper.

## Limitations

As a comparison of MBT and ETCM, an important and possibly crucial limitation was that patients tended to change case manager in the MBT arm. This introduced an important difference between the treatments, which was not related to the content, and nature of the therapy. It is of course important for patients with BPD for whom attachments are particularly problematic and for whom the ending of a relationship can and often does result in intense dysphoria and self-harm. As noted, we believe that this is the most likely factor explaining the higher rate of suicide attempts in the MBT compared with the ETCM arm of the study. The limitation emphasizes the need to keep all other aspects of treatment the same across treatment arms of future studies.

A second limitation was the method of defining SA vs. NSSH. This was done by a detailed assessment of contemporaneous records, but these records were not made primarily with the intent of determining or recording the level of suicidal intent associated with each episode of NSSH. However, this is a similar method to that used by Bateman and Fonagy (7). Each episode was assessed with the intention of determining intent, in order to make a risk assessment and this information was available in the electronic file. In the context of this service level trial, more specific assessment of the patients was not possible. The retrospective rating was done blind to the treatment. We therefore believe that while in the center of the spectrum of suicidality associated with self-harm, ratings are always relatively difficult, at either end of the spectrum, ratings are more reliable and that the distinction is useful and valid.

Thirdly, ethical approval included the requirement to provide participants in the control arm with the intervention following their participation in the study. This meant that a longer-term comparison of the two interventions was not possible.

Fourthly, access to primary care databases could not be obtained. This then meant that some participants could have presented to their general practitioner for treatment of NSSH/SA. However, self-harm of a serious nature would then have been referred to ED.

Finally, due to the almost total number of participants being female an examination of gender differences was not possible.

## Conclusion

The most similar previous RCT of MBT for BPD, Bateman and Fonagy (7) found a statistically significant advantage of MBT over manualised general treatment in rates of SA. They also demonstrated a superiority of MBT over SCM for NSSH, but only in the final 6 months. The current study was very similar to the Bateman and Fonagy study, but the results were obtained under “real-world” conditions in a public mental health service in another country, using non-research clinicians, relying on realistic levels of training, supervision and monitoring. Additionally, the subject population was drawn from existing caseloads with marked chronicity and severity as well as new referrals. What this study found was that either the introduction of a well-defined service that delivered a formal psychotherapy (MBT) or an effective structured case management approach (ETCM) resulted in significant reduction in SA and NSSH. The difference in improvements found between groups, across the two different outcomes (SA and NSSH) within this study setting will require further research.

## Data Availability Statement

The raw data supporting the conclusions of this article will be made available by the authors, without undue reservation.

## Ethics Statement

The studies involving human participants were reviewed and approved by Southern Regional Ethics Committee, Ministry of Health, NZ. The patients/participants provided their written informed consent to participate in this study.

## Author Contributions

DC: principle investigator and primary author contributing 60% of writing. RM: research mentor and fifth author contributing significant editing input. MC: research mentor and forth author contributing significant editing input. CF: statistical consultation and analysis. RP: final statistical analysis input and third author contributing 10% of writing. MI: primary statistical analysis. RG: co-investigator and secondary author contributing 20% of writing. All authors contributed to the article and approved the submitted version.

## Conflict of Interest

The authors declare that the research was conducted in the absence of any commercial or financial relationships that could be construed as a potential conflict of interest.
